# To relax restrictions: Are communities ready to deal with repeated epidemic waves of COVID-19?

**DOI:** 10.1017/ice.2020.228

**Published:** 2020-05-11

**Authors:** Jiancong Wang, Yew Fong Lee, Fangfei Liu, Mouqing Zhou

**Affiliations:** 1 Institute of Global Health, Faculty of Medicine, University of Geneva, Geneva, Switzerland; 2 Ministry of Health, Kuala Lumpur, Malaysia; 3 Dongguan Nosocomial Infection Control and Quality Improvement Center, Dongguan City, Guangdong Province, China

*To the Editor*—With strict lockdown and movement restriction measures, in Europe the incidence of newly confirmed COVID-19 cases has slowed down and the epidemiological curve has flattened.^1^ However, the World Health Organization (WHO) has warned that the peak of the pandemic has not yet passed.^2^ However, some countries are considering relaxing restrictions because they have to weigh ethical issues and social and economic crises against another potential COVID-19 wave.^3,4^ When and how to relax the restrictions have become items of strong debate between health politicians and other stakeholders. According to the WHO’s COVID-19 daily situation report and a recent study,^5,6^ community spread and clusters have predominantly contributed to most SARS-CoV-2 transmission. Therefore, the question facing policy makers remains: if restrictions are relaxed, will we be ready to deal with a repeated epidemic wave(s) in our community?

In China, resumption of works and production, reopening shops and restaurants, and even relaxation of travel restrictions have restored hope for virus-ravaged economies around the world.^7^ The National Health Commission of the People’s Republic of China strengthened and implemented various measures and/or policies in the face of another potential epidemic wave. Here, we summarize the key elements of infection prevention and control (IPC) measures implemented in China.

First, border control included screening and testing for COVID-19. Imported cases from abroad, especially international travelers, pose a potential threat to the community if they are not properly screened at the borders.^8^ According to data retrieved on the April 18, 9 of every 16 newly confirmed cases (56%) were identified as imported cases.^9^ Various measures (eg, travel history declaration, health epidemiological survey, temperature measurement, and rapid screening at airports) were conducted to efficiently detect suspected cases. All travelers were required to undergo a 14-day quarantine period at dedicated hotels, including SARS-CoV-2 testing by swab.^10^ Detected cases were directly referred to dedicated COVID-19 hospitals, which minimized the risk to close contacts and the spread of disease in the community.

Second, informative technology and the Health Declaration mobile telephone software application (ie, app)^11^ played a significant role in assessing the health status of residents. Information gathered was categorized and visualized using colored barcodes, which included each individual’s national identification number and address, temperature results (if available), 14-day travel history declaration, and contact history with suspected or confirmed COVID-19 patients. A green barcode indicated that a person was at low risk of having COVID-19 and/or transmitting SARS-CoV-2 and had been given approval for a “health permit” that allowed to access workplaces, shops, and restaurants (Supplementary Material Fig. 1 online). A red barcode indicated that a person was at high risk of having COVID-19 and/or transmitting SARS-CoV-2 and that he or she would be contacted by the local health authorities for mandatory quarantine measures (by law) and medical observation.

Third, China implemented ‘closed-off’ management of residential communities.^12^ Only community residents were permitted enter or exit their residential areas, and no visitors were allowed. Temperature measurement was mandatory upon entry, and mask wearing was compulsory upon exiting a residential area. Even though mask use is still being debated in some countries, Asia, Austria, Germany, and the Czech Republic have demonstrated positive effects of using masks in reducing further spread of SARS-CoV-2 in the community.^13^

Fourth, community and public healthcare services were reinforced and supported. In Guangzhou, Sun Yat-Sen Memorial Hospital launched online consultation services by a dedicated professional COVID-19 team via the “internet community hospital” platform.^14^ This online service provided timely and accessible healthcare services and information to residents in the community, thus avoiding hospital visits. Furthermore, the mental health of workers was also supported and monitored. In recent survey, 31.7% of respondents reported having fear when work resumed, and 28.6% of respondents reported having anxiety when work resumed.^15^ Local universities made mental health hotlines available to provide assistance, psychological consultation services, and even social support. For those with severe illness, psychological intervention by a specialist was recommended to minimize the impact of COVID-19 across the community.

Last but not least, the establishment of public health centers has been suggested to undertake in communities in first- and second-class cities. Their function includes providing medical supplies, as well as storage and distribution of medical products for emergency use (ie, masks, and disinfectants), not only for healthcare workers but also for local residents. We learned the lesson of a rapid increase in the demand for the medical products during early outbreak, and these facilities will quickly meet the medical needs of the community as well as reduce the risk of community spread of the virus.

In the latest press conference, WHO reiterated that although some countries have planned to relax restrictions due to socioeconomic concerns, the COVID-19 pandemic is not over in any country. Ending the COVID-19 pandemic requires continued efforts by individuals, communities, and governments to suppress and control this deadly virus. Finally, the WHO not only welcomes the accelerated advancement and implementation of SARS-CoV-2 antibody testing, which will help map infections in the community population; but will also provide technical, scientific, and financial support for sero-epidemiological investigations worldwide.

## Supplementary material

For supplementary material accompanying this paper visit http://dx.doi.org/10.1017/ice.2020.228. click here to view supplementary material
