# Supplementary material for: To relax restrictions: Are communities ready to deal with repeated epidemic waves of COVID-19?
Source: Infect Control Hosp Epidemiol. 2020 May 11:1–2. doi: 10.1017/ice.2020.228 (PMC7253764; doi:10.1017/ice.2020.228)

**Supplementary Material Fig. 1.** The green barcode of Health Declaration mobile software application. Note: This Health Declaration app platform is representative for the citizenship residing in Guangdong Province. The format of the Health Declaration app varies in different provinces. Information gathered are synchronized and shared across the country.

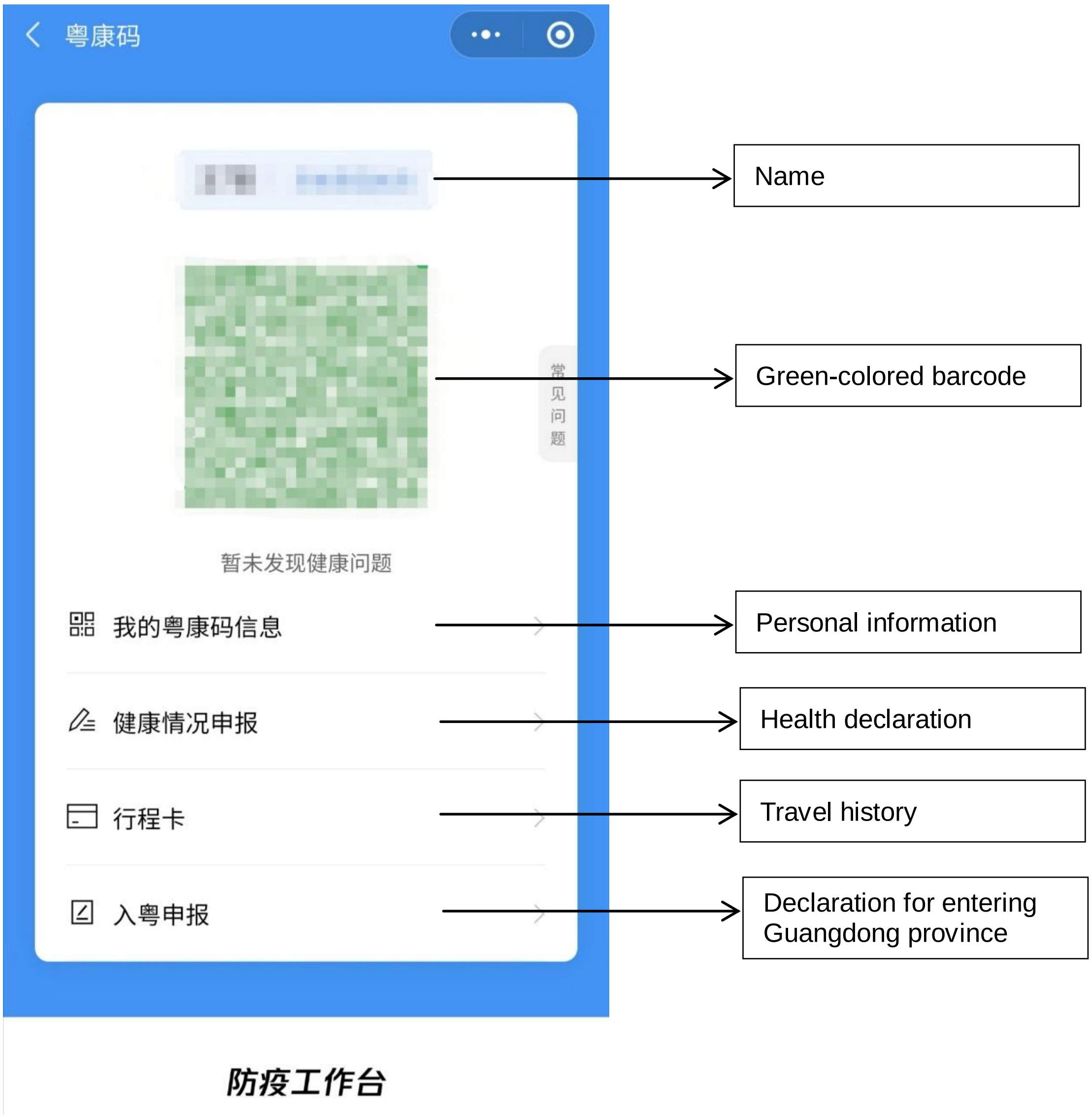

Supplement: Supplementary file 1 [file S0899823X20002287sup001.pdf]
